# Current Practices of Haemodynamic Monitoring in High-Risk Surgical Patients: A Nationwide Survey Among Malaysian Anaesthesiologists

**DOI:** 10.3390/healthcare13030339

**Published:** 2025-02-06

**Authors:** Syarifah Noor Nazihah Sayed Masri, Iskandar Khalid, Weng Ken Chan, Azarinah Izaham, Qurratu Aini Musthafa, Mohd Fitry Zainal Abidin, Siti Nadzrah Yunus, Ina Ismiarti Shariffuddin, Afifah Samsudin, Mohd Zulfakar Mazlan, Maxime P. Cannesson

**Affiliations:** 1 Department of Anaesthesiology & Intensive Care, Faculty of Medicine, Hospital Canselor Tuanku Muhriz, Universiti Kebangsaan Malaysia, Kuala Lumpur 56000, Malaysia; iskandarkhalid@gmail.com (I.K.); wengken@gmail.com (W.K.C.); azaizaham@ppukm.ukm.edu.my (A.I.); qurratuaini2112@gmail.com (Q.A.M.); 2 Department of Anaesthesiology, Faculty of Medicine, Universiti Malaya, Kuala Lumpur 50603, Malaysia; m.fitry@ummc.edu.my (M.F.Z.A.); siti.nadzrah@ummc.edu.my (S.N.Y.); ismiarti@ummc.edu.my (I.I.S.); 3 Department of Anaesthesiology & Intensive Care, Hospital Al-Sultan Abdullah UiTM, Puncak Alam 42300, Malaysia; afifahsamsudin@uitm.edu.my; 4 Department Anaesthesiology and Intensive Care, School of Medical Sciences, Universiti Sains Malaysia, Kubang Kerian 16150, Kelantan, Malaysia; zulfakar@usm.my; 5 Department of Anaesthesiology & Perioperative Medicine, David Geffen School of Medicine, Los Angeles, CA 90095, USA; mcannesson@mednet.ucla.edu

**Keywords:** perioperative goal-directed therapy, high-risk surgery, advanced haemodynamic monitoring, postoperative complications

## Abstract

Background: Advanced haemodynamic monitoring has been recommended for use in high-risk surgeries and high-risk patients undergoing surgery. This study aims to assess the current practices of haemodynamic monitoring in high-risk surgical patients among Malaysian anaesthesiologists. Methodology: This is a cross-sectional survey among Malaysian anaesthesiologists, following approval from the institution’s Medical Research Ethics Committee and the National Medical Research Register. The survey utilised a questionnaire developed by Cannesson et al. to gather demographic data, practice information, and haemodynamic monitoring practices. Statistical analysis was performed using SPSS, and results were presented as the mean, median, or frequency as appropriate. Results: A total of 366 participants responded to the questionnaire, and 2 dropped out due to an incomplete form. This study found differences in the frequency of haemodynamic optimisation and monitoring techniques used in different healthcare settings. Written protocols or statements concerning haemodynamic management in high-risk surgical cases were only available to 15.7% of participants in the institution. The overall utilisation rate of cardiac output monitoring was found to be 31.1%, with a significant majority of the usage observed in university hospitals (*p* < 0.001). Central venous pressure was more commonly used in university hospitals and private hospitals compared to public hospitals (*p* < 0.001). The usage of advanced parameters such as stroke volume variation, cardiac index, and systemic vascular resistance was significantly higher in university hospitals, with a *p* value < 0.001. Transthoracic echocardiography was the most common tool used for high-risk surgical patients. The primary reasons for participants not utilising cardiac output monitoring include the lack of availability of such monitoring in their respective settings, which constitutes 66.9% of the respondents. The overwhelming majority of participants, namely 98%, expressed the belief that there is room for improvement in their present haemodynamic care. Conclusions: This study offers significant insights into the prevailing haemodynamic monitoring practices employed by Malaysian anaesthesiologists in the context of high-risk surgical patients. The findings have the potential to contribute to future educational initiatives and establish practice standards for haemodynamic monitoring in high-risk surgical procedures.

## 1. Introduction

Advanced haemodynamic monitoring has been recommended for use in high-risk surgeries and in high-risk patients undergoing surgery. Numerous investigations have provided evidence supporting the view that perioperative haemodynamic optimisation has the potential to enhance postoperative outcomes among surgical patients who are at a higher risk for complications [1,2,3]. Haemodynamic optimisation in the context of high-risk surgical procedures has been shown to result in a reduction in postoperative complications as well as a decrease in the duration of both intensive care unit and hospital stays. Additionally, this approach has been associated with a reduction in the overall cost of the surgical procedure [3] and improved long-term survival [4].

Arterial hypotension is a frequent occurrence in patients undergoing surgery under general anaesthesia. Intraoperative hypotension has been demonstrated to jeopardise organ perfusion and is associated with postoperative morbidity and mortality [5]. Intraoperative hypotension (IOH) is often a late sign of haemodynamic compromise and is preceded by alterations in the cardiocirculatory state [6]. Several clinical studies have demonstrated an association between IOH and unfavourable effects on organ function and integrity (i.e., myocardial injury, stroke, and acute kidney injury) [7,8]. IOH is associated with an extended hospital length of stay, postoperative surgery-related morbidity, and even mortality [9]. The treatment of hypotension is highly dependent on its causes, invariably involving the administration of fluids (to optimise preload), vasopressors (to optimise afterload), and/or inotropes (to optimise contractility and thus cardiac output), along with the titration of the anaesthesia depth and compensating for surgery-related disturbances [10,11]. These interventions may cause an unfavourable increase in myocardial workload and oxygen requirement, which can be detrimental to susceptible patients.

The use of cardiac output (CO) measurements to guide fluid administration and inotropic therapy to optimise tissue perfusion and cellular oxygenation has been given the broad term goal-directed haemodynamic therapy (GDHT) [12]. Advancements in modern technology have brought about a variety of sophisticated monitors. Most of these newly developed techniques have enhanced our understanding of the mechanism of patient decompensation and have helped guide appropriate therapeutic interventions. Integrating these devices into therapeutic protocols enables the clinician to apply GDHT and rationally guide inotropic support and fluid administration, thereby reducing morbidity and mortality [13].

Despite the widespread interest in CO-guided haemodynamic treatment, there remains a lack of consensus about its adoption as the established standard of care for surgical patients at high risk [14,15]. Many practitioners do not routinely monitor CO and continue to use arterial blood pressure and central venous pressure (CVP) to guide haemodynamic optimisation in high-risk patients [13]. This strategy may also be due to the optimistic and erroneous assumption that arterial blood pressure and CO are closely related [16]. Most practitioners depend on blood pressure, CVP, and urine output as indicators of volume status. This practice continues despite consistent evidence demonstrating that CVP does not predict the fluid responsiveness or volume status of a patient [17].

Prior to the coronavirus (COVID) pandemic, there had been a gradual and steady increment in the total number of surgeries performed in Malaysia, with more than 1.8 million surgeries in 2016 alone [18]. Despite this, data on advanced haemodynamic CO monitoring usage in Malaysia remain elusive. Their use in the perioperative period is limited by cost, unfamiliarity, and a lack of knowledge in advanced haemodynamic monitoring. The objective of this survey was to evaluate the existing haemodynamic monitoring protocols employed in Malaysian hospitals for patients under high-risk surgical procedures. The primary aim of this study was to assess the prevalence of various haemodynamic monitoring techniques employed by anaesthesiologists in Malaysia for high-risk surgical procedures. We also sought to investigate the use of advanced haemodynamic monitoring as well as identify the barriers that hinder the adoption of advanced haemodynamic monitoring by anaesthesiologists in Malaysia for high-risk surgery.

## 2. Methodology

This study was approved by the Research and Ethics Committee of Universiti Kebangsaan Malaysia (JEP-2023-154) and the National Medical Research Register (NMRR ID-23-01913-8PE). This study was conducted in accordance with the principles outlined in the Declaration of Helsinki.

This study was conducted over a six-month period from 1 June 2023 to 31 December 2023. Participants were registered anaesthesiologist practicing in public, private, military, and university hospitals across Malaysia recruited via convenience sampling. Exclusion criteria included affiliate or retired anaesthesiologist and individuals who declined participation.

### 2.1. Study Questionnaire

We conducted a cross-sectional descriptive study using an online survey to assess the haemodynamic monitoring practices among anaesthesiologists in Malaysia. Data were collected using a questionnaire administered via Google Forms. The questionnaire was adapted and modified from a validated 34-item questionnaire by Cannesson et al. [13], designed to assess haemodynamic management and monitoring trends for high-risk surgical patients.

Twelve questions were related to respondents’ demographic information and clinical practices. The rest of the questions concerned respondents’ involvement in high-risk surgeries, areas of practice, availability of perioperative haemodynamic monitoring protocols, and specific haemodynamic monitoring techniques employed.

Eligible participants were invited through multiple channels: email through the Malaysian Society of Anaesthesiologist, an announcement at the Malaysian Society of Anaesthesiologists Annual Scientific Meeting, personal email, messages via social media platforms (e.g., WhatsApp), and emails disseminated by heads of anaesthesiology and intensive care departments. If no response was received after two weeks, a reminder was sent.

An English-language participant information sheet was included on the first page of the Google Form survey. Participation was voluntary, and submission of the completed questionnaire implied informed consent. No personal identification information was collected, ensuring participant confidentiality. Anaesthesiologists who did not respond after two reminder emails or within the study period were considered non-respondents. Incomplete questionnaires were classified as dropouts.

High-risk surgical patients were defined as patients with an increased risk of mortality, cardiac events, cerebrovascular accidents, or kidney impairment due to patient and/or surgical factors such as the following:

1. Cardiac or respiratory illness resulting in functional limitation.
2. Extensive surgery planned for carcinoma involving bowel anastomosis.
3. Predictable acute massive blood loss (>2.5 L).
4. Age over 70 years with functional limitations of one or more organ systems.
5. Septicaemia (positive blood cultures or septic focus).
6. Respiratory failure (PaO_2_ < 8 kPa on FiO_2_ > 0.4, i.e., PaO_2_/FiO_2_ ratio < 20 kPa or ventilation > 48 h).
7. Acute abdominal catastrophe (e.g., pancreatitis, perforated viscus, gastrointestinal bleed).
8. Acute renal failure (urea > 20 mmol/L, creatinine > 260 µmol/L).
9. Surgery for an abdominal aortic aneurysm.
10. Disseminated malignancy.
11. High-risk surgery with cardiovascular risk and a death rate of more than 5%.

### 2.2. Sample Size Calculations

The required sample size for this study was calculated using a standard formula for estimating proportions in cross-sectional studies. The calculation was based on prior research by Cannesson et al. [13], which reported that 34% of respondents used CO monitoring. A total of 362 participants were required to achieve 80% power for the study with a 95% confidence level and a 5% drop-out rate.

*n* = (Z^2^·P(1 − P))/d^2^)

where

*n* = required sample size;
Z = Z statistic for a 95% confidence level = 1.96;
P = prevalence = 0.34;
d = the degree of accuracy = 0.05.

Therefore,

*n* = (1.96^2^ × 0.34 × (1 − 0.34))/0.05^2^ ≈ 345

To account for a 5% drop out rate, the final sample size was adjusted to

*n* = 345 + (345 × 0.05) = 362

### 2.3. Statistical Test

All data analysis were performed using SPSS for Windows version 23.0 (IBM Corp., Armonk, NY, USA). Results were presented as mean ± standard deviation, median (interquartile range), or frequency (percentages) as appropriate. Categorical data were expressed as frequency. Data were analysed according to the number of responses obtained for each given question. The frequency distribution and two-way analysis were used to analyse categorical items. In all cases, two-tailed *p*-values of 0.05 or less were considered statistically significant.

## 3. Results

### 3.1. Demographic Data

**Figure 1:**
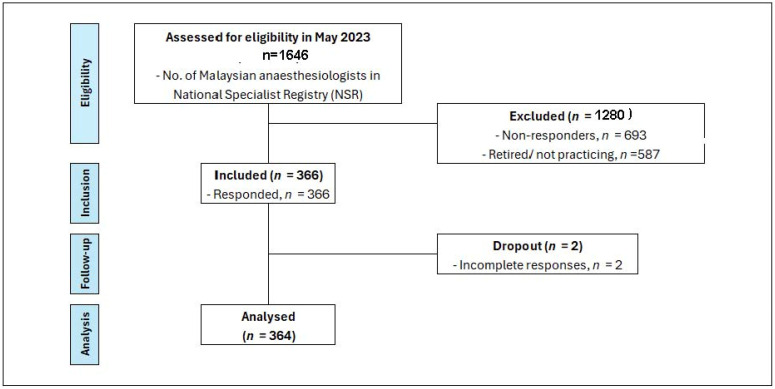
STROBE diagram—current practices of haemodynamic monitoring in high-risk surgical patients: a nationwide survey among Malaysian anaesthesiologists.

Figure 1 presents a STROBE diagram illustrating this study’s participant flow.

According to the National Specialist Register (NSR), as of May 2023, there were 1646 anaesthesiologists registered in Malaysia. Out of this number, 587 were retired or no longer practicing anaesthesiology. Among approximately 1059 of practicing anaesthesiologist, they were distributed across various healthcare sectors; approximately 60% practice in public hospitals, 30% in private hospitals, and 10% were affiliated with university hospitals. Three hundred and sixty-six (366) participants responded to the questionnaire, resulting in an overall response rate of 34.5%. After excluding 2 participants due to incomplete responses, the final dataset comprised 364 participants. The response rates from each sector were as follows: 37.7% (240 out of 635) from public hospitals, 21% (67 out of 318) from private hospitals, and 54% (57 out of 105) from university hospitals.

**Figure 2:**
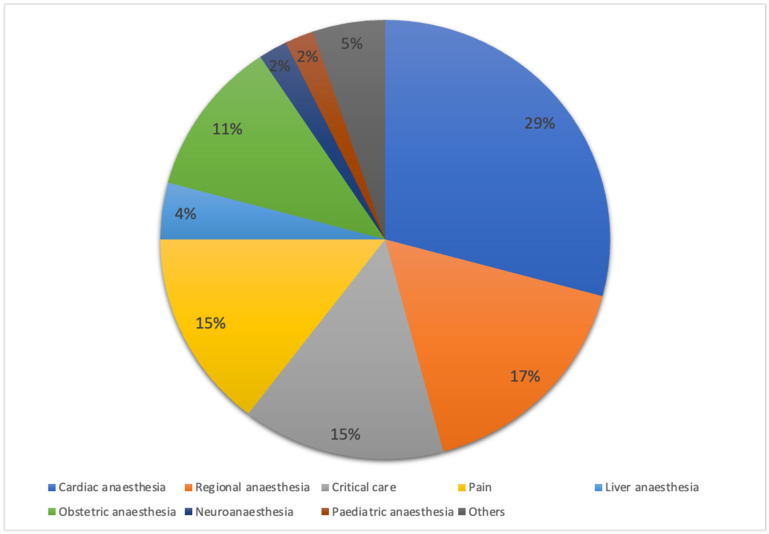
Distribution of respondents by subspecialty. Percentage distribution of respondents across subspecialties, including critical care, paediatric anaesthesia, and others. Data are expressed as percentages (%).

Of the total respondents, 347 (94.5%) provided anaesthesia for high-risk surgical patients. The majority managed high-risk surgical patients at an average of zero to five cases per week, with the highest proportion in university hospitals (80.7%), followed by public hospitals (76.3%) and private hospitals (75.5%), as highlighted in Table 1. Respondents in private hospitals had a median of 9.0 years of practice (interquartile range [IQR of 6.0–12.0 years), which was significantly longer (*p* < 0.001) compared to respondents in public hospitals (3.0 [2.0–7.0] years) and university hospitals (5.0 [3.0–8.8] years). Figure 2 illustrates the distribution of participants by subspecialty.

### 3.2. Types of Haemodynamic and Cardiac Output Monitoring and Techniques of Intraoperative Haemodynamic Optimisation

**Figure 3:**
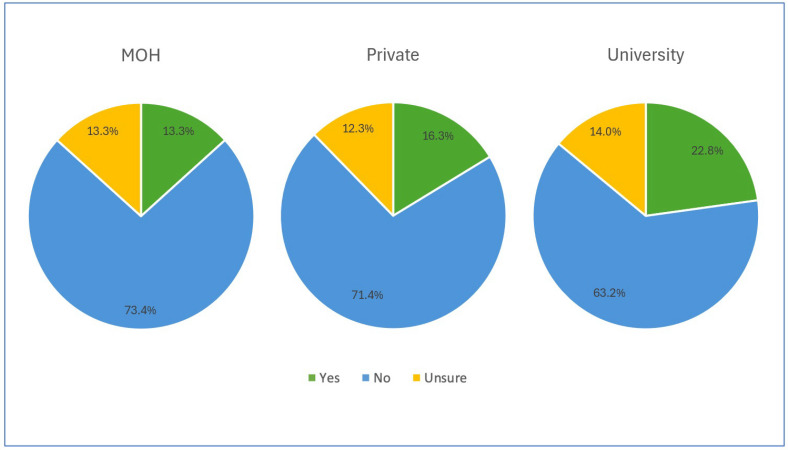
Availability of written protocol concerning haemodynamic monitoring among participants.

More than 60% of the participants in each institution reported the absence of written protocols concerning haemodynamic monitoring, and almost 12% were unsure of the existence of such protocols, as illustrated in Figure 3.

Table 2 summarises the types of routine haemodynamic monitoring used for managing high-risk surgeries among the respondents. The most commonly used haemodynamic monitoring modality in every institution was invasive arterial pressure (92.6%). The use of non-invasive arterial pressure as the main haemodynamic monitoring method was significantly lower in university hospitals (57.9%) compared to public hospitals (76.3%) and private hospitals (73.5%) (*p* = 0.022). Public hospitals showed a significantly lower use of CVP monitoring compared to private hospitals and university hospitals (32.1% vs. 67.3% vs. 63.2%; *p* < 0.001). On the other hand, CO or stroke volume (SV) (63.2%), stroke volume variation (SVV) (59.6%), and systemic vascular resistance (SVR) (57.9%) were the preferred modalities of haemodynamic monitoring in university hospitals, and these findings were statistically significant (*p* < 0.001). Both pulse pressure variation (PPV) and systolic pressure variation (SPV) usage were higher in public and university hospitals compared to private hospitals. PPV was used by 57.1% of respondents in public hospitals and 53.6% in university hospitals (*p* < 0.001), whereas SPV was used by 26.3% of university hospital respondents and 25.0% of public hospital respondents (*p* = 0.030). In addition, the use of near-infrared spectroscopy was more common in private hospitals (10.2%) and university hospitals (14.0%) than in public hospitals (2.5%), with this difference being statistically significant (*p* < 0.001).

On average, 75% of respondents performed haemodynamic optimisation before and after anaesthesia induction and during the operation across all institutions. However, in university hospitals, the proportion of haemodynamic optimisation performed during the postoperative period was lower (63.2%) compared to public (72.8%) and private hospitals (71.4%), though this difference was not statistically significant (*p* = 0.353).

According to the data presented in Table 3, most respondents conducted haemodynamic optimisation based on arterial pressure readings, with no significant difference in performance (*p* = 0.429). Haemodynamic optimisation based on dynamic parameters to evaluate fluid responsiveness was notably greater in university hospitals (91.2%), followed by public hospitals (85.8%) and private hospitals (69.4%) (*p* = 0.005). The practice of haemodynamic optimisation guided by CVP remained prevalent in university hospitals (86.0%) and private hospitals (73.5%). University hospitals had a considerably higher utilisation rate of haemodynamic optimisation based on central venous saturation of oxygen (ScvO_2_) and mixed venous saturation of oxygen (SvO_2_) compared to other hospitals (68.4% vs. 61.4%; *p* < 0.05).

In terms of CO monitoring (Table 4), 37.2% of the respondents used transthoracic echocardiography (TTE), with no significant difference among institutions (*p* = 0.097). A notable percentage of respondents expressed a preference for utilising an EV1000/Hemosphere, with usage rates of 22.4% in public hospitals, 18.3% in private hospitals, and 63.2% in university hospitals (*p* < 0.001). Transoesophageal echocardiography (TOE) was also preferred by some respondents, with 6.4% usage in public hospitals, 18.4% in private hospitals, and 26.3% in university hospitals (*p* = 0.003).

### 3.3. Fluid Responsiveness

**Figure 4:**
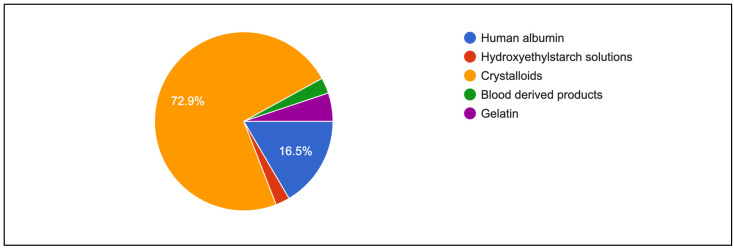
First choice of fluid resuscitation among respondents.

Figure 4 shows primary intravenous resuscitation of participants. Crystalloids (72.9%) and human albumin (16.5%) were mainly used as primary intravenous fluid for resuscitation. Table 5 shows indicators and the assessment of fluid responsiveness among respondents. Blood pressure remained a common method for monitoring response to volume expansion (79.0%), although it was less preferred in university hospitals (63.2%; *p* < 0.001). More than 65% of respondents in every institution observed an increase in blood pressure as a response to volume expansion (*p* = 0.025). A similar preference was found for using urine output as an indicator of volume expansion, and an increase in urine output was routinely observed. Both findings were statistically significant (*p* = 0.021). In addition, a significant proportion of respondents in public hospitals and university hospitals used PPV as a measure of volume expansion (65.1% vs. 59.6%; *p* < 0.001). However, only a quarter of the respondents considered SV (23.5%) and SVV (24.3%) as the best predictors of a rise in CO following volume expansion. Further subgroup analysis also showed that more senior anaesthesiologists tend to rely on central venous pressure rather than dynamic parameters such as systolic pressure variation and pulse pressure variation (Table 6).

### 3.4. Participants Perception

Overall, 31% of respondents routinely used advanced haemodynamic monitoring or CO monitoring in high-risk surgical patients. The main hindrance was the unavailability of equipment in their institutions, experienced by up to 70% of respondents in public and private hospitals, compared to 35.1% in university hospitals (35.1%; *p* < 0.001) (Table 7). As a substitute for CO, a large portion of respondents in both public and university hospitals (40.2% and 40.4%, respectively; *p* = 0.039) utilised dynamic parameters of fluid responsiveness, such as changes in pulse pressure, systolic pressure, and plethysmographic waveforms. Overall, 98.0% of respondents expressed a positive attitude toward the further improvement of haemodynamic management in the future.

## 4. Discussion

This study provides a comprehensive analysis of haemodynamic practices among anaesthesiologists in Malaysia and identifies obstacles to achieving GDHT in high-risk surgical procedures. We categorised our survey respondents into three primary types of institutions in Malaysia: Ministry of Health (MOH) hospitals (public hospitals), which are publicly funded; privately owned hospitals (private hospitals); and university-affiliated hospitals (university hospitals). The majority of our respondents were from public hospitals.

The vast majority of respondents (96.4%) were directly engaged in supervising high-risk surgeries in their clinical practice. However, only about 30% of those practicing in public and private hospitals incorporated advanced haemodynamic monitoring into their clinical procedures. This finding closely aligns with the 2011 study by Cannesson et al., which reported that only 34% of anaesthesiologists in Europe and the United States monitored CO in similar situations [13]. In contrast, anaesthesiologists in university hospitals exhibited a considerably higher utilisation rate of advanced parameters, including SV, SVV, and CO, at approximately 60% during high-risk surgical procedures.

Despite the well-documented benefits of monitoring and optimising CO to improve outcomes during high-risk surgeries, the majority of institutions lacked established protocols for haemodynamic monitoring. Only a mere 15% of institutions had established written protocols for its implementation. This percentage is significantly lower compared to Japanese and Chinese institutions, where 27.6% and 26% of respondents’ institutions, respectively, reported having protocols related to haemodynamic monitoring [19,20]. This discrepancy may be a result of the smaller proportion of major specialist hospitals in Malaysia, which account for approximately 19.5% of all Malaysian hospitals, where high-risk surgeries are more likely to be performed, thus necessitating the use of established written protocols [21].

University hospitals demonstrated a notably greater adoption rate of GDHT compared to public and private hospitals. Specifically, 63.2% of respondents from university hospitals reported regularly utilising SV and CO measurements. Additionally, 57.9% and 59.6% of respondents reported using SVR and SVV, respectively. On the other hand, less than 25% in private hospitals utilised more advanced parameters for GDHT. This stark difference in adoption rates between university hospitals and private hospitals highlights potential disparities in resources, and institutional priorities across different healthcare settings.

Our study indicates that haemodynamic optimisation by the majority of anaesthesiologists in Malaysia occurs both prior to and following anaesthesia induction, as well as during surgery. However, the rate of haemodynamic optimisation decreases to around 70% in the postoperative period. It is important to acknowledge that the occurrence of haemodynamic instability after surgery can affect up to 31% of patients over a 24 h period, significantly increasing the risk of mortality and morbidity [22]. Ensuring optimal haemodynamics throughout the postoperative period is, therefore, equally crucial for patients undergoing high-risk surgeries.

The higher frequency of CVP monitoring in university and private hospitals compared to public hospitals in Malaysia is consistent with global trends observed in surveys involving members of the Korean Society of Anaesthesiologists (KSA), the American Society of Anesthesiology (ASA), and the European Society of Anesthesiology (ESA) [13,23]. This difference in practice is likely due to factors such as resource availability, clinical experience, and institutional guidelines. University and private hospitals often have greater access to advanced monitoring technologies and training, which likely influences their decision to incorporate CVP in routine haemodynamic management. In contrast, public hospitals, where resources and infrastructure may be more limited, may rely more on basic monitoring tools like blood pressure and urine output, reflecting a greater reliance on clinical experience and the availability of simpler resources [13]. These findings align with the KSA survey, which showed frequent use of CVP as an indicator of fluid status despite its limited predictive value for fluid responsiveness [23]. While CVP has traditionally been used as a marker of preload and fluid responsiveness, its limitations have been emphasised by recent studies, which note that CVP can be influenced by various factors, including blood volume, intrathoracic pressure, and venous compliance, making it an unreliable standalone indicator [24,25,26]. Despite these limitations, CVP remains a valuable safety measure in more resource-intensive settings, such as university and private hospitals, where it is often combined with dynamic measures of fluid responsiveness as recommended by the institutional protocols [27].

University hospitals also demonstrated a greater frequency of optimising advanced parameters such as SV, CO, ScvO_2_, and dynamic parameters in assessing fluid responsiveness. Many studies have shown that implementing proactive monitoring and intervention strategies using GDHT during high-risk surgeries significantly contributes to maintaining blood flow and oxygen perfusion, ultimately leading to improved recovery, reduced complication rates, reduced length of stay, and reduced mortality [28].

The CO monitoring techniques used in Malaysian hospitals include LiDCO, oesophageal Doppler, PiCCO, TTE, an EV1000 or Hemosphere monitor, and an ultrasonic cardiac output monitor (USCOM). Among these, the EV 1000 is used significantly more in university hospitals. In contrast, public and private hospital respondents utilised TTE more frequently. TTE is commonly used because it does not involve the use of expensive disposable components and has a straightforward learning curve. GDHT, using echocardiography, is a preferred technique in intensive care settings [29,30]; however, its use in the intraoperative setting is less convenient and does not allow for the continuous monitoring of haemodynamic conditions [31]

A small number of respondents indicated that the monitors were not easily available at their individual facilities. Specifically, this was reported by 12.9% of respondents in public hospitals, 16.3% in private hospitals, and 1.8% in university hospitals. According to Chen G et al., approximately one-third of assessed hospitals in China lacked access to pulse contour analysis equipment (such as the Vigileo or LiDCO monitors), thoracic bioimpedance monitors, and oesophageal Doppler monitors [20]. We hypothesise that hospitals without CO monitoring are those that rarely conduct high-risk procedures at their facility or lack intensive care facilities.

Despite the widespread availability of CO monitoring technology, the majority of our respondents refrained from using it in the perioperative management of high-risk surgical patients. Prior systematic reviews have demonstrated the efficacy of GDHT in reducing postoperative complications after major surgeries [3]. The reluctance to employ advanced haemodynamic monitoring may stem from the equipment unavailability in respective hospitals, as reported by 70% of participants in public hospitals and 75% in private hospitals. As a result, they resort to PPV or SPV as surrogates for CO. In addition, the utilisation of haemodynamic monitoring is impeded by the knowledge, skills, and perceptions of anaesthesiologists. Notably, 10% of participants lack familiarity with CO monitor usage, while a subset (ranging from 2.0% to 3.7%) perceive it as devoid of new clinically significant information in perioperative scenarios.

It is interesting to note that in managing volume expansion in the perioperative setting, a significant majority of anaesthesiologists in public hospitals (88.5%) and private hospitals (65%) used urine output and blood pressure as their main markers, incorporating their clinical experience into the patients’ volume management. In contrast, the majority of anaesthesiologists in university hospitals employed CO (54.4%) and SVV (59.6%) to guide volume expansion management in perioperative patients. Multiple studies have demonstrated that intraoperative oliguria is not only caused by a decrease in fluid volume; it is also influenced by sympathetic activity and hormonal factors. Rectifying oliguria does not necessarily enhance outcomes [32]. Permissive oliguria has demonstrated benefits in the perioperative context [33].

Interestingly, we discovered that more senior anaesthesiologists employ CVP as a monitoring tool in high-risk surgery, whereas younger generations use more dynamic indicators such as PPV and SPV. Previous haemodynamic research has not compared age differences with anaesthesiologists’ preferences for utilising a tool in a high-risk procedure. Suehiro et al., however, discovered a general trend in CVP being less frequently monitored than dynamic indicators [19]. A study on anaesthesiologists in a university hospital in South Africa found that anaesthetists with 5–10 years of experience usually utilised PPV as an indicator of volume response [34].

This study holds particular importance as it represents the first comprehensive survey of haemodynamic monitoring practices in high-risk surgery within Malaysia’s healthcare landscape. By venturing into previously unexplored areas, the study not only fills a critical knowledge gap but also lays the foundation for future advancements in patient care. The findings provide valuable insights into current practices and highlight areas where resource allocation, training, and protocol development could enhance patient outcomes.

The survey on haemodynamic monitoring in Malaysia is crucial, despite the acknowledged low usage rates, as it provides localised data that can inform focused interventions. As a developing nation, the primary barrier to employing haemodynamic monitoring is the expense associated with disposables and the necessity to acquire new skills and data analysis capabilities. We are confident that, despite the low utilisation, most respondents are eager to learn new technologies and adopt them for improved patient care. Understanding the specific challenges faced by Malaysian anaesthesiologists—related to resources or education—can guide the development of tailored training programmes and practices. This report highlights the need for improved patient safety practices in high-risk surgical situations, suggesting that even minor adjustments in monitoring methods could significantly benefit patient outcomes. This localised information can enable policy adjustments and improve clinical procedures within the Malaysian healthcare system.

### Limitations

This study has several limitations that may impact the findings. Firstly, the overall response rate was low, with only 34.2% of eligible anaesthesiologists participating. This raises concerns about non-response bias, as those who did not participate might have different practices or opinions, potentially limiting the generalisability of the results.

Secondly, while we included anaesthesiologists from public, private, and university hospitals, the sample was not proportionally representative of the national distribution of anaesthesiologists across different sectors. There was an over-representation of respondents from university hospitals and an under-representation of public and private hospitals. Several factors may have contributed to this imbalance. University hospital anaesthesiologists may have greater involvement in research activities and easier access to academic surveys. In contrast, anaesthesiologists working in public and private hospitals might face heavier clinical workloads or have less engagement with academic research, resulting in lower response rates. This uneven representation could include our findings and may lead to an overestimation or underestimation of advanced haemodynamic monitoring utilisation across different sectors.

Additionally, the reliance on an electronic survey introduces potential biases, such as ascertainment bias, favouring anaesthesiologists who are more comfortable with technology or more engaged with online professional networks. This may have excluded those less technologically inclined, further impacting the diversity of responses.

Furthermore, the use of convenience sampling rather than random sampling limits the ability to generalise the results to the entire population of Malaysian anaesthesiologists. Future studies employing stratified random sampling and multiple survey methods could help achieve a more representative sample.

We also did not include the protocol available in the questionnaire; hence, we were unable to identify if the protocols were based on dynamic parameters, fluid responsiveness strategies, or goal-directed therapy using lactate, urine output, or mean arterial pressure as the primary endpoint. Including these types of questions could help provide more comprehensive insights into the clinical protocols being used and whether they align with current best practices in perioperative goal-directed therapy.

Finally, this was a single-point study; we were not able to carry out a pre and post evaluation following recent publications. We were not able to capture the impact of recent publications given the nature of our study. In future, a longitudinal design would allow for a more robust analysis of how protocols impact the outcome. By comparing data before and after intervention, we could better assess their effectiveness or identify areas for improvement.

## 5. Conclusions

This study sheds light on the current haemodynamic monitoring practices used by Malaysian anaesthesiologists in the setting of high-risk surgical patients. The adoption of perioperative CO monitoring in high-risk surgery in Malaysia remains relatively low, with only 31% of respondents routinely utilising these methods. The primary barriers identified include equipment unavailability and lack of standardised protocols. The findings from this study could guide future training programmes and help set practice standards for haemodynamic monitoring in high-risk surgical procedures in Malaysia. By enhancing resource allocation, developing institutional guidelines, and promoting targeted education, the adoption of advanced haemodynamic monitoring can be increased to improve patient outcomes in high-risk surgical procedures.

## Figures and Tables

**Table 1:** Demographic data and background haemodynamic management among participants.

Variables	All Participants (N = 346)	Public Hospital (N = 240)	Private Hospital (N = 49)	University Hospital (N = 57)	*p* Value
Years of practice, median [IQR]		3.0 [2.0–7.0]	9.0 [6.0–12.0]	5.0 [3.0–8.8]	<0.001 † a. <0.001 b. 0.001 c. 0.001
Participant that has subspecialty	45 (12.3)	17 (7.1)	15 (30.6)	13 (22.8)	<0.001 †
No. of hospital beds					
<250 beds	58 (15.8)	24 (10.0)	30 (61.2)	4 (7.0)	<0.001 †
251–1000 beds	214 (58.5)	156 (65.0)	18 (36.7)	40 (70.2)	
>1000 beds	74 (20.2)	60 (25.0)	1 (2.0)	13 (22.8)	
No. of estimated population					
<50,000	52 (14.2)	34 (14.8)	8 (16.7)	10 (18.9)	0.636
50,001–500,000	168 (45.9)	123 (53.7)	21 (43.8)	24 (45.3)	
>500,000	110 (30.1)	72 (31.4)	19 (39.6)	19 (35.8)	
Frequency of providing anaesthesia for a high-risk patient					
0 to 5 times a week	266 (72.7)	183 (76.3)	37 (75.5)	46 (80.7)	0.751
More than 5 times a week	80 (21.9)	57 (23.8)	12 (24.5)	11 (19.3)	

Data were expressed n (%). † Statistically significant *p* value < 0.05, overall comparison among three groups. a. Comparison between public and private hospital; b. comparison between public and university hospitals; c. comparison between private and university hospitals. *p* value < 0.0167, post hoc test with Bonferroni correction.

**Table 2:** Types of routine haemodynamic monitoring used for the management of high-risk surgery in different institutions.

Routine Haemodynamic Monitoring Use for the Management of High-Risk Surgery Patients				
Institution	Public Hospital (N = 240)	Private Hospital (N = 49)	University Hospital (N = 57)	*p* Value
Central venous pressure	77 (32.1)	33 (67.3)	36 (63.2)	<0.001 *
Stroke volume variation	62 (25.8)	11 (22.4)	34 (59.6)	<0.001 *
Pulse pressure variation	137 (57.1)	12 (24.5)	30 (53.6)	<0.001 *
Near-infrared spectroscopy	6 (2.5)	5 (10.2)	8 (14.0)	<0.001 *
Cardiac output/stroke volume	60 (25.0)	12 (24.5)	36 (63.2)	<0.001 *
Systemic vascular resistance (SVR)	49 (20.4)	10 (20.4)	33 (57.9)	<0.001 *
Non-invasive arterial pressure	183 (76.3)	36(73.5)	33 (57.9)	0.022 *
Systolic pressure variation	60 (25.0)	4 (8.2)	15 (26.3)	0.030 *
Invasive arterial pressure	234 (97.5)	48 (98.0)	57 (100.0)	0.711
Plethysmographic waveform variation	56 (23.3)	10 (20.4)	13 (22.8)	0.906
Global end-diastolic volume	8 (3.3)	4 (8.2)	5 (8.8)	0.090
Mixed venous saturation (SvO_2_)	19 (7.9)	3 (6.1)	8 (14.0)	0.293
Central venous saturation (ScvO_2_)	40 (16.7)	6 (12.2)	7 (12.3)	0.577
Oxygen delivery (DO_2_)	7 (2.9)	2 (4.1)	4 (7.0)	0.307
Pulmonary capillary wedge pressure	8 (3.3)	1 (2.0)	6 (10.5)	0.050
Oesophageal Doppler monitor	0 (0)	0 (0)	0 (0)	-

Data are expressed as numbers and percentages. * Statistical significance with a *p* value < 0.05.

**Table 3:** Haemodynamic optimisation strategy and management options during the perioperative period.

Institution	All Participants (N = 346)	Public Hospital (N = 240)	Private Hospital (N = 49)	University Hospital (N = 57)	*p* Value
Optimise haemodynamics					
Before anaesthesia induction	301 (82.2)	214 (88.8)	37 (75.5)	50 (87.7)	0.043 *
After anaesthesia induction	280 (76.5)	194 (80.5)	40 (81.6)	46 (80.7)	0.983
During surgery	286 (78.1)	198 (82.5)	43 (87.8)	45 (78.9)	0.487
Postoperative period	245 (66.9)	174 (72.8)	35 (71.4)	36 (63.2)	0.353
Do you optimise arterial pressure intraoperatively?					
No	3 (0.8)	2 (0.8)	1 (2.0)	0 (0.0)	0.429
Yes	343 (93.7)	238 (99.2)	48 (98.0)	57 (100.0)	
Do you optimise central venous pressure?					
No	126 (34.4)	105 (43.8)	13 (26.5)	8 (14.0)	<0.001 *
Yes	220 (60.1)	135 (56.3)	36 (73.5)	49 (86.0)	
Do you optimise stroke volume and/or cardiac output?					
No	111 (30.3)	84 (35.0)	20 (40.8)	7 (12.3)	0.002 *
Yes	235 (64.2)	156 (65.0)	29 (59.2)	50 (87.7)	
Do you optimise central venous oxygen saturation (ScvO_2_)?					
No	175 (47.8)	126 (52.5)	31 (63.3)	18 (31.6)	0.003 *
Yes	171 (46.7)	114 (47.5)	18 (36.7)	39 (68.4)	
Do you optimise mixed venous oxygen saturation (SvO_2_)?					
No	198 (54.1)	143 (59.6)	33 (67.3)	22 (38.6)	0.005 *
Yes	148 (40.4)	97 (40.4)	16 (32.7)	35 (61.4)	
Do you optimise dynamic parameters of fluid responsiveness?					
No	54 (17.8)	34 (14.2)	15 (30.6)	5 (8.8)	0.005 *
Yes	292 (79.8)	206 (85.8)	34 (69.4)	52 (91.2)	

Data are expressed as numbers and percentages. * Statistical significance with *p* < 0.05.

**Table 4:** Techniques used to monitor cardiac output.

	Technique Used To Monitor Cardiac Output				
Institution	All Participants (N = 346)	Public Hospital (N = 240)	Private Hospital (N = 49)	University Hospital (N = 57)	*p* Value
LiDCO Monitor	24 (6.6)	17 (7.1)	0 (0.0)	7 (12.3)	0.059
Swan–Ganz	24 (6.6)	13 (5.4)	4 (8.2)	7 (12.3)	0.201
PiCCO monitor	81 (22.1)	56 (23.2)	10 (20.4)	15 (26.3)	0.837
Transesophageal echocardiography	40 (10.9)	16 (6.4)	9(18.4)	15(26.3)	0.003 *
Transthoracic echocardiography	136 (37.2)	103 (42.7)	16 (32.7)	17 (29.8)	0.097
EV1000/Hemosphere	99 (27.0)	54 (22.4)	9 (18.3)	36 (63.2)	<0.001 *
USCOM	16 (4.3)	12 (5.0)	3 (6.1)	1 (1.8)	0.545
Arterial line waveform	3 (0.8)	2 (0.8)	1 (2.0)	0 (0.0)	0.452
Clinical	6 (1.6)	3 (1.2)	3 (6.1)	0(0.0)	0.084
Not Available	40 (10.9)	31 (12.9)	8 (16.3)	1 (1.8)	0.029 *

Data are expressed as numbers and percentages (%). * Statistical significance with *p* < 0.05.

**Table 5:** Indicators and assessment of fluid responsiveness among respondents.

Institution	All Participant (N = 346)	Public Hospital (N = 240)	Private Hospital (N = 49)	University Hospital (N = 57)	*p* Value
What are your indicators for volume expansion in this setting (diagnostic tools)? (please mark all that apply)					
Central venous pressure	113 (30.9)	67 (27.8)	20 (40.8)	26 (45.6)	0.015 *
Central venous saturation (SvO_2_)	34 (9.3)	23 (9.5)	4 (8.2)	7 (12.3)	0.755
Urine output	276 (75.4)	200 (83.0)	38 (77.6)	38 (66.7)	0.021 *
Cardiac output	141 (38.5)	95 (39.4)	15 (30.6)	31 (54.4)	0.036
Transesophageal echocardiography	47 (12.8)	28 (11.6)	8 (16.3)	11 (19.3)	0.259
Mixed venous saturation ScvO_2_	18 (4.9)	11 (4.6)	2 (4.1)	5 (8.8)	0.384
Pulse pressure variation or Systolic pressure variation	209 (57.1)	157 (65.1)	18 (36.7)	34 (59.6)	0.001 *
Stroke volume variation	150 (41.0)	100 (41.5)	16 (32.7)	34 (59.6)	0.012 *
Pulmonary capillary wedge pressure	11 (3.0)	3 (1.2)	3 (6.1)	5 (8.8)	0.056
Plethysmographic waveform variation	44 (12.0)	36 (14.9)	6 (12.2)	2 (3.5)	0.066
Global end-diastolic volume	24 (6.6)	15 (6.2)	3 (6.1)	6 (10.5)	0.484
Clinical experience	223 (61.9)	155 (64.3)	35 (71.4)	33 (57.9)	0.350
Blood pressure	289 (79.0)	213 (88.4)	40 (81.6)	36 (63.2)	0.001 *
How do you routinely assess the haemodynamic effects of volume expansion in this setting? (please mark all that apply)					
Increase in urine output	262 (71.6)	187 (77.6)	40 (81.6)	35 (61.4)	0.021*
Increase in cardiac output	163 (44.5)	110 (45.6)	23 (46.9)	30 (52.6)	0.636
Decrease in stroke volume	146 (39.9)	94 (39.0)	16 (32.7)	36 (63.2)	0.001 *
Decrease in pulse pressure	210 (57.4)	156 (64.7)	22 (44.9)	32 (56.1)	0.027 *
Increase in blood pressure	277 (75.7)	199 (82.6)	40 (81.6)	38 (66.7)	0.025 *
Increase in mixed ScvO_2_	16 (4.4)	9 (3.7)	3 (6.1)	4 (7.0)	0.372
Decrease in heart rate	270 (73.8)	196 (81.3)	36 (73.5)	38 (66.7)	0.042
Decrease in plethysmographic	69 (18.8)	52 (21.6)	10 (20.4)	7 (12.3)	0.285
Increase in central venous saturation	24 (6.6)	14 (5.8)	4 (8.2)	6 (10.5)	0.346
In your opinion, which parameter best predicts an increase in cardiac output following volume expansion?					
Central venous pressure	1 (0.3)	0 (0.0)	1 (2.0)	0 (0.0)	-
Mixed venous saturation (ScvO_2_)	1 (0.3)	0 (0.0)	0 (0.0)	1 (2.0)	
Global end-diastolic volume	19 (5.2)	13 (5.4)	4 (8.2)	2 (3.5)	
Stroke volume variation	89 (24.3)	62 (25.7)	11 (22.4)	16 (28.1)	
Transesophageal echocardiography	32 (8.7)	25 (10.4)	4 (8.2)	3 (5.3)	
Pulse pressure variation	42 (11.5)	33 (13.7)	3 (6.1)	6 (10.5)	
Central venous saturation (ScvO_2_)	1 (0.3)	1 (0.4)	0 (0.0)	0 (0.0)	
Plethysmographic waveform variation	3 (0.8)	2 (0.8)	1 (2.0)	0 (0.0)	
Cardiac output	86 (23.5)	61 (25.3)	10 (20.4)	15 (26.3)	
Clinical experience	20 (5.5)	14 (5.8)	4 (8.2)	2 (3.5)	
Pulmonary capillary wedge pressure	10 (2.7)	5 (2.1)	2 (4.1)	3 (5.3)	
Blood pressure	39 (10.7)	25 (10.4)	8 (16.3)	6 (10.5)	

Data are expressed as numbers and percentages (%). * Statistical significance with *p* < 0.05.

**Table 6:** Type of hemodynamic monitoring with years of practice.

Hemodynamic Monitoring	<5 Years of Practice (*n* = 187)	5–10 Years of Practice (*n* = 105)	>10 Years of Practice (*n* = 54)	^∫^ *p* Value	^‡^ *p* Value
Non-invasive arterial pressure	134 (71.7)	80 (76.2)	38 (70.4)	0.639	
Invasive arterial pressure	184 (98.4)	102 (97.1)	53 (98.1)	0.869	
Plethysmographic waveform variation	37 (19.8)	23 (21.9)	19 (35.2)	0.057	
Global end-diastolic volume	7 (3.7)	6 (5.7)	4 (7.4)	0.494	
Central venous pressure	64 (34.2)	46 (43.8)	36 (66.7)	<0.001	*p* value ^a^ = 0.086 *p* value ^b^ = 0.005 *p* value ^c^ = 0.269
Stroke volume variation	57 (30.5)	31 (29.5)	19 (35.2)	0.751	
Mixed venous saturation (SvO_2_)	17 (9.1)	8 (7.6)	5 (9.3)	0.899	
Central venous saturation (ScvO_2_)	31 (16.6)	15 (14.3)	7 (13.0)	0.761	
Oxygen delivery (DO_2_)	7 (3.7)	5 (4.8)	1 (1.9)	0.739	
Pulse pressure variation	119 (63.6)	44 (41.9)	16 (29.6)	<0.001	*p* value ^a^ = <0.001 *p* value ^b^ = <0.001 *p* value ^c^ = <0.001
Near-infrared spectroscopy	11 (5.9)	5 (4.8)	3 (5.6)	0.922	
Pulmonary capillary wedge pressure	6 (3.2)	4 (3.8)	5 (9.3)	0.166	
Transesophageal echocardiography	0 (0.0)	0 (0.0)	0 (0.0)	-	
Systolic pressure variation	55 (29.4)	16 (15.2)	8 (14.8)	0.007	*p* value ^a^ = <0.001 *p* value ^b^ = <0.001 *p* value ^c^ = 0.102
Cardiac output/stroke volume	54 (28.9)	32 (30.5)	22 (40.7)	0.248	
Systemic vascular resistance (SVR)	46 (24.6)	29 (27.6)	17 (31.5)	0.577	

Data are expressed in frequency (percentage). ^∫^ *p* value < 0.05, overall comparison among three groups; ^‡^ *p* value < 0.0167, post hoc test with Bonferroni correction; ^a^, comparison between <5 and 5–10; ^b^, comparison between <5 and >10; ^c^, comparison between 5–10 and >10.

**Table 7:** Reasons for respondents not utilising cardiac output monitoring in high-risk surgery.

If You Do Not Monitor Cardiac Output Routinely in These Patients, What Are the Main Reasons for Not Monitoring It? (Please Mark All That Apply)					
Institutions	All Participant (N = 346)	Public Hospital (N = 240)	Private Practice (N = 49)	University Hospital (N = 57)	*p* Value
I use SvO_2_ and/or ScvO_2_ as surrogates for cardiac output monitoring	16 (4.4)	10 (4.1)	0 (0.0)	6 (10.5)	0.075
I use dynamic parameters of fluid responsiveness (Pulse Pressure Variations, Systolic Pressure Variations, Plethysmographic Waveform Variations) as surrogates for cardiac output	132 (36.1)	97 (40.2)	12 (24.5)	23 (40.4)	0.039 *
Available cardiac output monitoring solutions are too invasive	39 (10.7)	28 (11.6)	7 (14.3)	4 (7.0)	0.514
Cardiac output monitoring equipment not readily available	227 (62.0)	170 (70.5)	37 (75.5)	20 (35.1)	<0.001 *
Cardiac output monitoring does not provide any additional clinically relevant information in this setting	12 (3.3)	9 (3.7)	1 (2.0)	2 (3.5)	1.000
Cost	12 (3.3)	6 (2.5)	1 (2.0)	5 (8.8)	0.140
Unfamiliarity in utilising cardiac output monitoring	36 (9.8)	26 (10.8)	5 (10.2)	5 (8.8)	0.980
I monitor cardiac output routinely	2 (0.5)	0 (0.0)	1 (2.0)	1 (1.8)	0.317
Clinical evaluation & fluid responsiveness	1 (0.3)	0 (0.0)	0 (0.0)	1 (1.8)	0.317
Available cardiac output monitoring solutions are unreliable	7 (1.9)	6 (2.5)	1 (2.0)	0 (0.0)	0.744

* Statistical significance with *p* < 0.05.

## Data Availability

The data are available at https://doi.org/10.6084/m9.figshare.28075346.v1.
